# Exploration and validation of a combined immune and metabolism gene signature for prognosis prediction of colorectal cancer

**DOI:** 10.3389/fendo.2022.1069528

**Published:** 2022-11-28

**Authors:** Yitai Xiao, Guixiong Zhang, Lizhu Wang, Mingzhu Liang

**Affiliations:** ^1^ Guangdong Provincial Key Laboratory of Biomedical Imaging and Guangdong Provincial Engineering Research Center of Molecular Imaging, The Fifth Affiliated Hospital, Sun Yat-sen University, Zhuhai, Guangdong, China; ^2^ Department of Interventional Oncology, The First Affiliated Hospital, Sun Yat-sen University, Guangzhou, Guangdong, China; ^3^ Department of Radiology, The Fifth Affiliated Hospital, Sun Yat-sen University, Zhuhai, Guangdong, China

**Keywords:** colorectal cancer, immune, prognosis, metabolism, gene signature

## Abstract

**Background:**

Colorectal cancer (CRC) is still one of the most frequently diagnosed malignancy around the world. The complex etiology and high heterogeneity of CRC necessitates the identification of new reliable signature to identify different tumor prognosis, which may help more precise understanding of the molecular properties of CRC and identify the appropriate treatment for CRC patients. In this study, we aimed to identify a combined immune and metabolism gene signature for prognosis prediction of CRC from large volume of CRC transcriptional data.

**Methods:**

Gene expression profiling and clinical data of HCC samples was retrieved from the from public datasets. IRGs and MRGs were identified from differential expression analysis. Univariate and multivariate Cox regression analysis were applied to establish the prognostic metabolism-immune status-related signature. Kaplan-Meier survival and receiver operating characteristic (ROC) curves were generated for diagnostic efficacy estimation. Real-time polymerase chain reaction (RT-PCR), Western blot and immunohistochemistry (IHC) was conducted to verified the expression of key genes in CRC cells and tissues.

**Results:**

A gene signature comprising four genes (including two IRGs and two MRGs) were identified and verified, with superior predictive performance in discriminating the overall survival (OS) of high-risk and low-risk compared to existing signatures. A prognostic nomogram based on the four-gene signature exhibited a best predictive performance, which enabled the prognosis prediction of CRC patients. The hub gene ESM1 related to CRC were selected *via* the machine learning and prognostic analysis. RT-PCR, Western blot and IHC indicated that ESM1 was high expressed in tumor than normal with superior predictive performance of CRC survival.

**Conclusions:**

A novel combined MRGs and IRGs-related prognostic signature that could stratify CRC patients into low-and high- risk groups of unfavorable outcomes for survival, was identified and verified. This might help, to some extent, to individualized treatment and prognosis assessment of CRC patients. Similarly, the mining of key genes provides a new perspective to explore the molecular mechanisms and targeted therapies of CRC.

## Introduction

Accounting for 10.9% of all cancers in men and 9.5% of the all cancers in women, colorectal cancer (CRC) is still one of the most frequently diagnosed malignancy around the world (1). Despite remarkable advances in early diagnosis and management within the past decades, the prognosis for CRC patients remains unsatisfactory (2). Even though new therapeutic options such as immunotherapy and targeted therapy have been explored with certain success, the average 5-year survival probability for patients with advanced CRC is still discouraging (3). The complex etiology and high heterogeneity of CRC necessitates the identification of new reliable signature to identify different tumor prognosis, which may help more precise understanding of the molecular properties of CRC and identify the appropriate treatment for CRC patients.

In addition to the classic tumor, node, metastasis (TNM) staging, several molecular features unique to CRC, such as microsatellite instability (MSI), chromosomal instability (CIN) and CpG island methylator phenotype (CIMP), provide indispensable guidance for tailored treatment as well as prognostic assessment (4). For instance, CRC patients with a low proportion of KRAS mutations shall be more likely to benefit from epidermal growth factor receptor (EGFR) antibody therapy (5), while patients with MSI-H molecular profiles do not receive an overall survival advantage from immune checkpoint-blockade administration (6). These findings suggested that immunotherapy is likely to be effective against specific subtypes of CRC. Recent findings revealed that immune alterations, which was used for molecular subtypes of low-grade diffuse glioma, were correlated with different immune subtypes, manifesting as different lymphocyte profiles, tumor mutation load and clinical regression (7). This inspired us that some signatures, especially immune-related signatures, can be utilized for molecular stratifying of CRC to develop personalized treatment strategies and evaluation of clinical survival outcome.

Emerging evidences have suggested an inextricable connection between tumor growth and metabolic pathways (8). The differences in metabolic patterns between tumor cells and normal cells enable tumor cells to exhibit unique metabolic profiles of glucose, fatty acids and amino acids (9). As one of the hallmarks of CRC, metabolism reprogramming due to various causes leads to metabolic interactions between immune cells, cancer stem cells, the tumor microenvironment (TME) and the gut microbiota, ultimately resulting in diverse therapeutic responses and clinical outcomes (10). Such metabolic differences are expected to be a promising anti-cancer strategy, as in-depth exploration of the molecular changes caused by metabolism rewiring could facilitate the advancement of targeted therapies (11).

In this study, we aim to identify a combined immune and metabolism gene signature for prognosis prediction of CRC. A four-gene signature based on immune-related genes (IRGs) and metabolism-related genes (MRGs) from large volume of CRC transcriptional data was identified and validated. This signature will facilitate a deeper understanding of the molecular mechanisms of immunity and metabolism in CRC and provide guidance for more precise personalized immunotherapy.

## Materials and methods

### Data source

The mRNA expression profiles and corresponding clinical information associated with CRC patients were obtained from TCGA-COAD, including 473 tumor and 41 normal tissue samples) and GSE38832 dataset (https://www.ncbi.nlm.nih.gov/geo/query/acc.cgi?acc=gse38832, including 118 CRC tissue samples).The RNA-sequencing data (containing clinical and molecular information) in TCGA COAD project were downloaded from the Genomic Data Commons (GDC) Data Portal (https://portal.gdc.cancer.gov/) and gene microarray dataset containing CRC samples were downloaded from Gene Expression Omnibus (GEO) database (https://www.ncbi.nlm.nih.gov/geo/). Four datasets containing pair samples (GSE113513, GSE74602, GSE44076, GSE41328) were obtained to identify differentially expressed genes (DEGs) from GEO. GSE113513 included 14 pairs of cancerous and matched non-cancerous tissues. GSE74602 consisted of 30 pairs of normal and tumor tissue samples from patients with CRC. GSE44076 contained 98 pairs of colon tumor and adjacent normal mucosal tissue samples. GSE41328 comprised 10 pairs of CRC and matched normal colon tissue samples. Duplicate samples and samples without key clinical features or survival information were excluded. A total of 2752 MRGs were collected from The Molecular Signature Database (MSigDB) (https://www.gsea-msigdb.org/gsea/msigdb/index.jsp) while 2483 IRGs were downloaded from the ImmPort database (https://immport.niaid.nih.gov).

### Construction of an individualized prognostic signature

A Weighted Gene Co-Expression Network Analysis (WGCNA) was conducted to mine the coexpressed genes and modules in CRC according to the gene expression profiles by the R package “WGCNA” (12). Briefly, a gene co-expression network was constructed and then the samples were clustered using hierarchical clustering. In order to identify the modules of interest, the correlation between each coexpression module and CRC samples was further evaluated. Modules with significant correlation with the CRC samples were defined as key modules for the subsequent selection of hub genes.

The DEGs between CRC tumor tissues and normal tissues were performed using the “limma” R package with an adjusted *P* value< 0.05 and |log2FC| > 1 being set to identify significant DEGs (13).

The univariate Cox regression analysis using the R package “survival” was conducted to identify the prognostic value of these DEGs for overall survival (OS) and genes with *P* values less than 0.05 were considered statistically significant. To avoid overfitting, a least absolute shrinkage and selection operator (LASSO) Cox regression analysis was conducted. Subsequently, a combined metabolism- and immune-related signature was formulated through the multivariate Cox regression. The risk scores were calculated followed by the formula: $RiskScore=\sum_{i=1}^{n}\left(Gene\;Expressioni\times Coefi\right)$ . In order to standardize and normalize riskScore, the risk index was introduced and calculated as follows: riskIndex = (riskScore-min)/(max-min).

CRC patients were divided into high-risk and low-risk groups according to the median riskIndex. Kaplan-Meier survival curves was plotted to evaluate of the prognosis between different groups. The receiver operating characteristic (ROC) curve was constructed using R package “survivalROC” to evaluate the efficacy of the risk model. The R package “stats” and “Rtsne” were applied to conduct principal component analysis (PCA) and t-distributed stochastic neighbor embedding (t-SNE) to assess the clustering of the signature genes.

Univariate and multivariate Cox regression analyses of clinical pathology were conducted to identify potential risk factors for overall survival in the TCGA cohort. A nomogram plot was constructed to predict the 1-, 2-, 3-, 5-, and 10-year OS rate by incorporating riskScores and clinical characteristics with the R package “rms”. The calibration curves were used to estimate the fitting degree of the established nomogram model. The predictive performance of the nomogram was subsequently evaluated using the time-dependent ROC analysis. Decision Curve Analysis (DCA) was employed to evaluate the efficacy of using the complex model as a decision-maker tool.

### Tumor microenvironment analysis

The infiltrating score of 17 immune cells and the activity of 13 immune-related pathways were further calculated with single-sample Gene Set Enrichment Analysis (ssGSEA) applying the R package “gsva”. Immune and stromal scores were further estimated to quantify the immune and stromal components by the ESTIMATE algorithm using the R package “ESTIMATE”.

#### Selection of characteristic genes *via* machine learning algorithms

After filtration of differentially expressed genes in GSE41328, GSE44076 and TCGA datasets, the candidate hub genes related to CRC were selected *via* the SVM-RFE (Support Vector Machine-Recursive Feature Elimination) algorithm searching for lambda with the smallest classification error to determine the variable. SVM-RFE was applied for feature selection *via* ten-fold cross-validation. ROC curves and the area under the ROC curve (AUC) were used for estimating the diagnostic efficacy.

### Real-time polymerase chain reaction (RT-PCR)

Normal intestinal epithelial cell line FHC and human colorectal adenocarcinoma cell line (LS-174T, RKO, SW-620, HT-29, and HCT-116) were obtained from the American Type Culture Collection (ATCC). The cells were cultured in DMEM: F­12 medium (FHC) or RPMI-1640 medium (other cell lines) containing with 10% fetal bovine serum (FBS). Total RNA was extracted from the cells with FastPure^®^ Cell/Tissue Total RNA Isolation Kit (#RC112, Vazyme, Nanjing, China) according to the manufacturer’s instruction. Approximate 1000 ng of RNA was used to for cDNA synthesis by PrimeScript RT reagent Kit (#RR037A, Takara Bio, Kyoto, Japan). RT-PCR was performed using SYBR Green Mix (#4309155, Thermo Fisher Scientific, USA). Gene expression was standardized to the expression of GADPH. Primer sequences are as follows: GAPDH-F: G TGG TCT CCT CTG ACT TCA ACA; GAPDH-R: C TCT TCC TCT TGT GCT CTT GCT; ESM1-F: TG TTT CCT ATG CCC CAG AAC; ESM1-R: GC CCT TCC TTG GTA GGT AGC.

### Western blot

The cells and tissues were collected and then lysed with radio-immunoprecipitation assay (RIPA, Beyotime, Shanghai, China) buffer containing a protease inhibitor mixture. The supernatant was collected and the protein concentrations were qualified by a BCA Protein Quantitation Kit (Thermo Fisher Scientific, Waltham, USA). Equal amounts of protein were separated by sodium dodecyl sulfate polyacrylamide gel electrophoresis (SDS-PAGE) on a 10% polyacrylamide gel and transferred onto a PVDF membrane. The membranes were blocked with 5% skim milk and incubated with primary antibody against ESM1 (bs-3615R, Bioss, Beijing, China) or GAPDH (10494-1-AP, Proteintech, Chicago, USA) overnight at 4°C. The next day, after washing thrice with PBST (phosphate buffered saline with Tween 20), the membranes were incubated horseradish peroxidase (HRP)-conjugated secondary antibody (#7074, Cell Signaling Technology, Danvers, USA). The protein bands were eventually visualized using enhanced chemiluminescence (ECL) substrate (Genesion, Guangzhou, China) and imaged by a chemiluminescence system (Bio-Rad, Hercules, USA).

### Immunohistochemistry (IHC)

Tumor sections was obtained from post-surgery specimens with an informed consent waiver. Postoperative tumor tissues derived from patients were fixed in 4% paraformaldehyde and embedded in paraffin. The tissues were sectioned into 5-μm slices and deparaffinized with xylene followed by rehydrated with graded alcohols. Antigen retrieval was performed in a boiling pressure cooker with citrate buffer (pH = 6.0) for 10 min. The sections were incubated with anti-ESM1 antibody (bs-3615R, Bioss, Beijing, China) at a dilution of 1:200 overnight at 4°C. After washing with PBST the next day, the sections were incubated with an HRP-conjugated anti-rabbit secondary antibody (MaxVision, Fuzhou, China) for half an hour at room temperature. Finally, the sections were visualized with diaminobenzidine (DAB, ZSGB Bio, Beijing, China) and counterstained with hematoxylin. The IHC images were captured by a slide Scanner System (3DHISTECH, Budapest, Hungary) and Immunohistochemistry scores (H-scores) were quantified by 3DHISTECH QuantCenter software (3DHISTECH, Budapest, Hungary). Since there is no universally accepted standard, we considered a score below 50 as negative expression, 50-100 as weak positive, 100-150 as medium positive, and greater than 150 as strong positive based on previous literature (14).

### Statistical analysis

All data analyses were carried out using R software (version 4.1.1, https://www.r-project.org). Student’s *t*-test or Wilcoxon’s rank-sum test was used for the comparison of continuous variables while Pearson’s χ^2^ test or Fisher’s exact test was used for the comparison of categorical variables. Experimental data were presented as means ± standard deviation (SD) and statistical analysis was conducted by GraphPad Prism Software (version 9.1, GraphPad, San Diego, USA). A two-tailed *P*-value less than 0.05 was considered statistically significant.

## Results

### Identification and enrichment analysis of IRGs and MRGs

**Figure 1:**
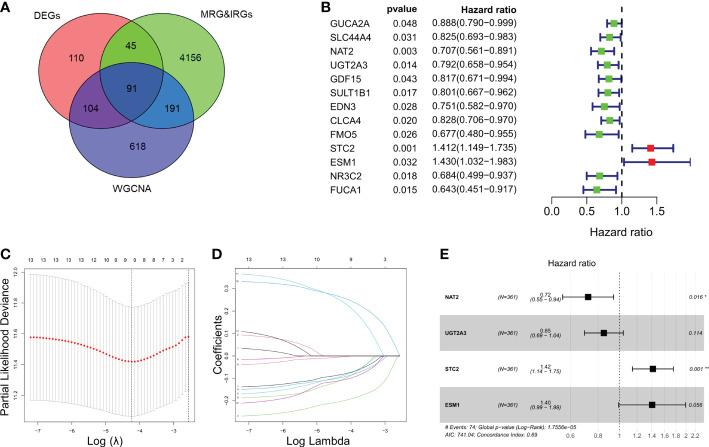
Identification and enrichment analysis of metabolism-related genes (MRGs) and immune-related genes (IRGs). **(A)** Venn diagram of 91 differentially expressed MRGs and IRGs intersections. **(B)** Univariate Cox regression analysis of the relationship between in different genes and OS. **(C)** Cross-validation for tuning parameter selection using LASSO Cox regression. **(D)** Coefficient profiles in the LASSO Cox regression model. **(E)** Forest plots of univariate Cox regression analysis of different gene expression and the corresponding OS. *P < 0.05, **P < 0.01.

In this study, we included a total of 361 CRC patients from TCGA (as a training set) and 118 patients from GSE38832 (as a validation set). A total of 350 genes and 1004 genes were identified by DEGs and WGCNA analysis respectively among CRC patients (**Figure S1A, B**). Total of 91 intersected MRGs and IRGs were extracted from DEGs and WGCNA analysis (**Figure 1A**). Among them, 13 significantly differentially expressed genes were significantly associated to the prognosis of CRC, while two genes, STC2 and ESM1, were significantly correlated with a poor prognosis of CRC (**Figure 1B**). LASSO penalized Cox regression was conducted to reduce overfitting of 13 genes. A stepwise multivariate Cox regression analysis was entered and 4 genes were eventually selected to generate an optimal prognostic signature (**Figures 1C–E**). Respective coefficient values were extracted to determine risk scores using the following formula: RiskScore = NAT2 × (-0.32727) + UGT2A3 × (-0.16282) + STC2 × (0.34818) + ESM1 × (0.33961). RiskIndex = (riskScore-min)/(max-min).

### Validation of the prognostic signature

**Figure 2:**
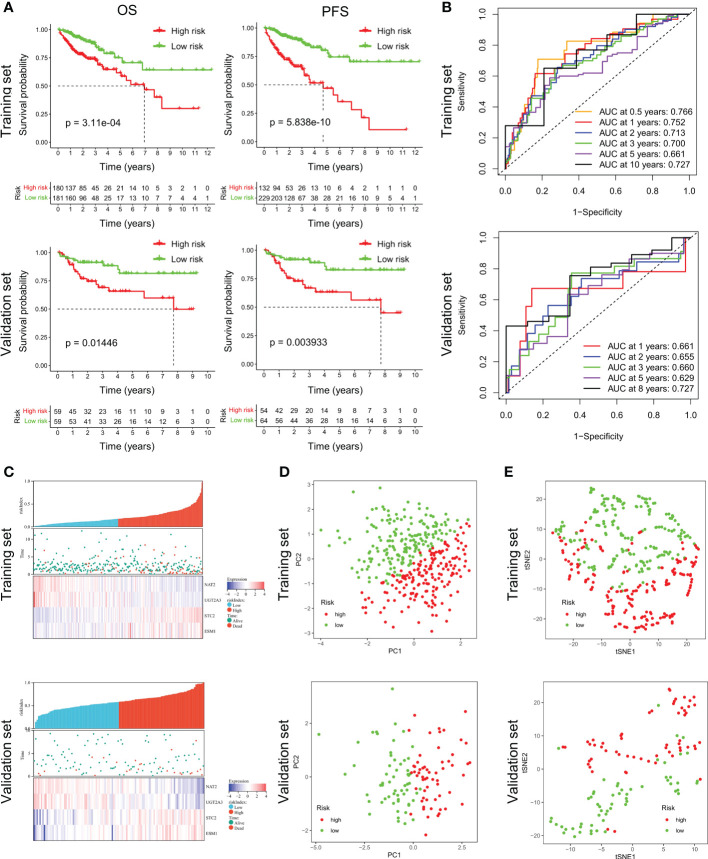
Survival analysis of CRC patients in the training and validation datasets. **(A)** Respective Kaplan-Meier OS and PFS curves in the training and validation datasets. **(B)** Time-dependent ROC curves for CRC patients at the time points of 0.5, 1, 2, 3, 5 and 10 (or 8) years. **(C)** RiskScore distribution, survival status, and expression heatmap of the selected four genes in the high- or low-risk groups. **(D, E)** PCA and t-SNE analysis confirmed the clustering of combined metabolism- and immune-related signature.

According to the median riskIndex, all of the CRC patients were divided equally into low-risk and high-risk groups. As illustrated in **Table 1**, members of the different groups were significantly correlated with TNM stage (*P*< 0.05). Survival analysis indicated that high-risk group exhibited a significantly worse progression-free survival (PFS) and OS than low-risk group, either in the training set or validation set (**Figure 2A**). To evaluate the predictive value of the constructed signature, the time-dependent ROC curve analysis was performed and the AUC of 1, 2, 3, 5 and 10 (or 8) years were 0.766, 0.752, 0.713, 0.700, 0.661, 0.727 in TCGA cohort and 0.661, 0.655, 0.660, 0.629, 0.727 in GSE38832 cohort, respectively (**Figure 2B**). As riskIndex distribution curve, survival status, and expression heatmap of the signature shown in **Figure 2C**, patients with high riskIndex experienced higher mortality and higher expression of the STC and ESM1 and lower expression of the NAT2 and UGT2A3 both in the training set and validation set. PCA (**Figure 2D**) and t-SNE (**Figure 2F**) analysis in the training set or validation set confirmed the risk profile differences between low- and high-risk groups. Thus, the combined metabolism- and immune-related signature exhibited superior performance for prediction of the survival and progression of CRC.

**Table 1:** Baseline characteristics of the patients in TCGA cohort.

Variables	Group	TCGA cohort (n = 361)		*P* value
		High risk (n = 180)	Low risk (n = 181)	
Median survival time (days)		677.5	735	
Survival status	Alive	129 (71.67%)	158 (87.29%)	< 0.001
	Dead	51 (28.33%)	23 (12.71%)	
Gender	Female	80 (44.44%)	87 (48.07%)	0.4902
	Male	100 (55.56%)	94 (51.93%)	
Age (years)	≤ 60	53 (29.44%)	54 (29.83%)	0.9333
	> 60	127 (70.56%)	127 (70.17%)	
TNM stage	I	21 (11.67%)	42 (23.20%)	0.0052
	II	68 (37.78%)	76 (42.00%)	
	III	56 (31.11%)	41 (22.65%)	
	IV	35 (19.44%)	22 (12.15%)	
T	1	2 (1.11%)	6 (3.31%)	0.0036
	2	20 (11.11%)	42 (23.21%)	
	3	132 (73.34%)	118 (65.19%)	
	4	26 (14.44%)	15 (8.29%)	
N	0	95 (52.78%)	120 (66.30%)	0.0031
	1	43 (23.89%)	42 (23.20%)	
	2	42 (23.33%)	19 (10.50%)	
M	0	145 (80.56%)	159 (87.85%)	0.0575
	1	35 (19.44%)	22 (12.15%)	

### Prognostic value of the gene signature

**Figure 3:**
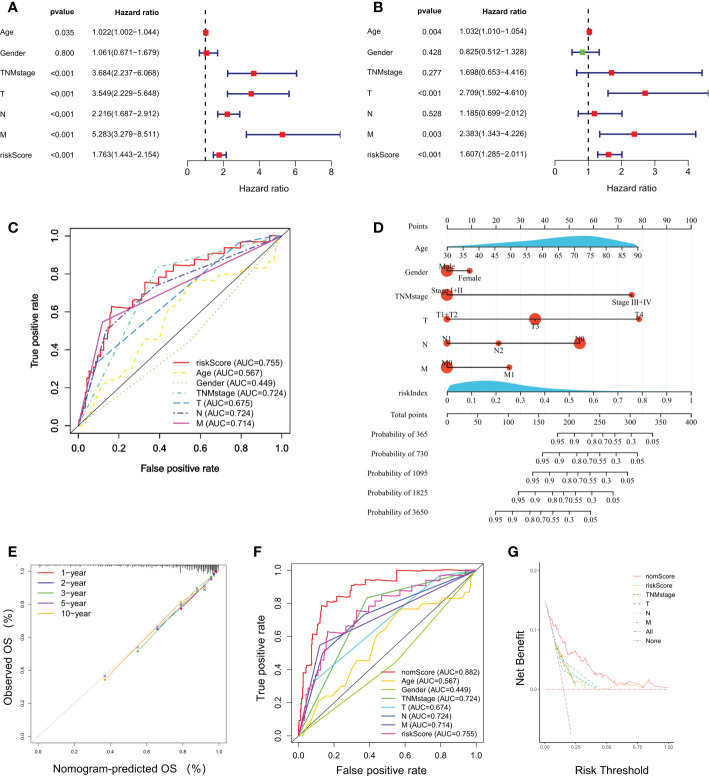
Independent prognostic power of the selected four-gene signature. **(A, B)** Univariate and multivariate Cox regression analyses of the relationship between different clinical parameters and riskScore with OS. **(C)** Evaluation of the prognostic utility of the riskScore and clinical parameters using ROC curves. **(D)** Nomogram comprised the riskIndex and clinical parameters for predicting the prognosis probability in CRC. **(E)** Calibration curves of the nomogram showed consistency in the predicted and observed 1-, 2-, 3-, 5 and 10-year survival rates. **(F)** ROC curve analysis of the nomogram for OS. **(G)** Decision curve analysis (DCA) of the nomogram compared with other parameters.

Univariate and multivariate Cox regression analyses indicated that riskScore was significantly correlated to a poor prognosis of CRC in TCGA cohort (**Figures 3A, B**). The ROC curves for CRC patients revealed that the AUC of riskScore to predict OS were 0.755, higher than age, gender, and TNM stage (**Figure 3C**). Based on riskScore and clinicopathological factors such as age, gender, and TNM stage, a prognostic nomogram was constructed to predict the survival rate of CRC patients (**Figure 3D**). The calibration curves demonstrated good concordance between predicted and actual 1-, 2-, 3-, 5- and 10- year survival rates, which indicated an excellent performance of the prognostic nomogram (**Figure 3E**). The ROC curve analysis showed a nomogram AUC of 0.882, which was significantly higher than other parameters, such as riskScore, age, gender, and TNM stage (**Figure 3F**). A DCA was applied to evaluate the prognostic nomogram, which ranked as the highest in the net benefit accompanied with a broader range of threshold probability among all the parameters (**Figure 3G**). These results suggested that the prognostic nomogram exhibited a best predictive performance and was more suitable for predicting the prognosis of CRC patients in clinical practice.

### Construction and validation a nomogram based on riskIndex and TNM stage

**Figure 4:**
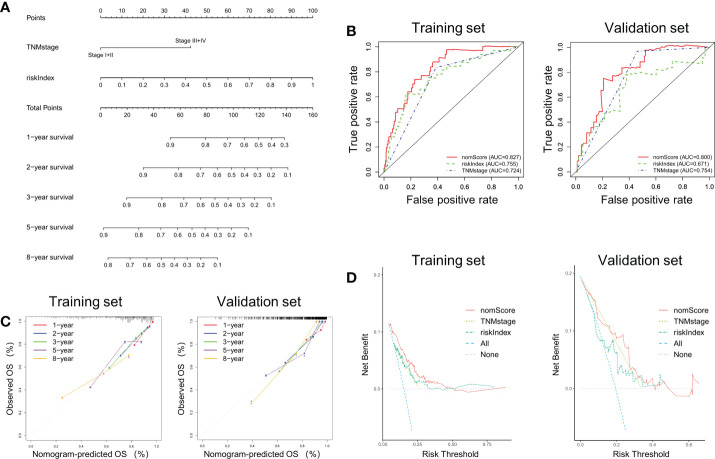
Nomogram based on riskIndex and TNM stage for external validation. **(A)** Nomogram comprised the riskIndex and TNM stage for predicting the prognosis probability in CRC. **(B)** ROC curves for comparison of the nomogram, riskIndex and TNM stage in the training and validation datasets. **(C)** Calibration curves of the nomogram to predict the 1, 2, 3, 5 and 10-year survival rates. **(D)** DCA of the integrated nomogram in the training and validation datasets.

According to the previous analysis it can be seen that riskIndex and TNM stage greatly affect the prognosis of CRC patients. Given that the external validation set GSE38832 has only TNM stage as a clinical parameter, a prognostic nomogram based on riskIndex and TNM stage was generated to predict the survival of CRC patients in TCGA cohort and verified in GSE38832 cohort (**Figure 4A**). The nomogram AUCs of ROC curves were 0.827 and 0.800, which was significantly higher than riskIndex and TNM stage either in the training set or validation set, respectively (**Figure 4B**). The calibration curves revealed good consistency between predicted and actual 1-, 2-, 3-, 5- and 8- year survival rates, which indicated an excellent performance of the nomogram (**Figure 4C**). The DCA showed that the nomogram ranked as the highest in the net benefit accompanied with a broader range of threshold probability among other parameters (**Figure 4D**).

**Figure 5:**
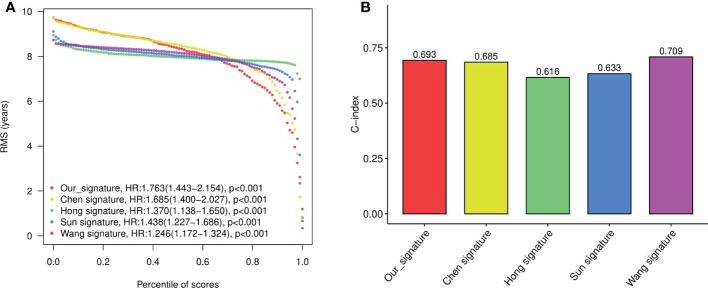
The performance of the constructed four-gene signature compared to previous signatures. **(A)** The restricted mean survival time (RMST) curves for each signature obtained by integrating signatures. **(B)** C-index (concordance index) for each signature obtained by integrating signatures.

To evaluate the performance of the signature against existing signatures, four published risk models for OS in CRC patients were included for comparison (15–18). As shown by Kaplan-Meier curve analysis, our four-gene model, as well as all four other models, showed significant prognostic value of CRC in predicting OS (**Figure S2A**). The ROC of each signatures revealed that all the models exhibited good predictive performance, with the AUC at 1-, 2-, 3-, and 5- year larger than 0.6 (**Figure S2B**). Restricted mean survival time (RMST) showed that although our model has a slightly lower C-index (concordance index) than Wang’s model and higher than other models, our four-gene signature held the highest hazard ratio (HR) among all the gene signatures (**Figures 5A, B**).

### Immunological features annotation of the signature

**Figure 6:**
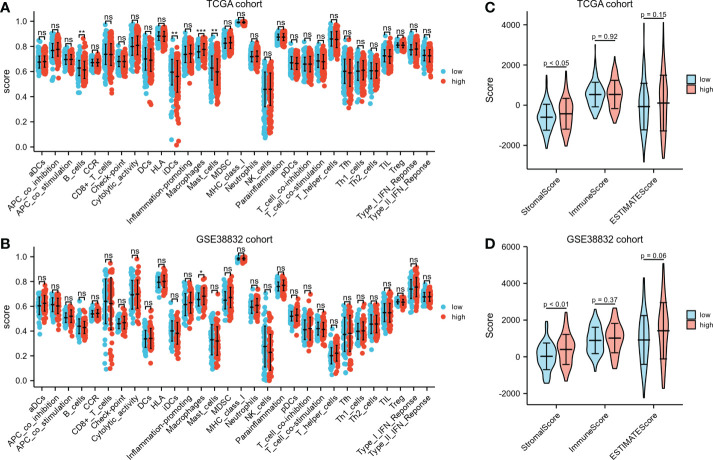
Immunological features annotation of the signature. **(A, B)** The infiltration levels of immune cell components between the two risk groups in TCGA CRC cohort and GSE38832 cohort. **(C, D)** Stromal score, immune score and ESTIMATE score calculated by ESTIMATE algorithm in TCGA CRC cohort and GSE38832 cohort. *P < 0.05, **P < 0.01, ***P < 0.001, ns, no significance.

Different immunocyte infiltration between the two risk groups based on ssGSEA was exhibited in **Figures 6A, B**. Noteworthy, regardless in TCGA cohort or GSE38832 cohort, higher percentages of macrophages were observed in high-risk group than low-risk group. ESTIMATE algorithm was applied to compare the differences of immunocyte infiltration between the high- or low-risk groups. The high-risk group showed significantly higher stromal score compared with the low-risk group either in TCGA cohort or GSE38832 cohort (*P*< 0.05, **Figures 6C, D**). These results indicated that CRC patients with high-risk scores have more abundant stromal components in the tumor microenvironment, which may lead to a worse prognosis in the high-risk group due to greater susceptibility to metastasis.

### Validation of the role of ESM1 in CRC

**Figure 7:**
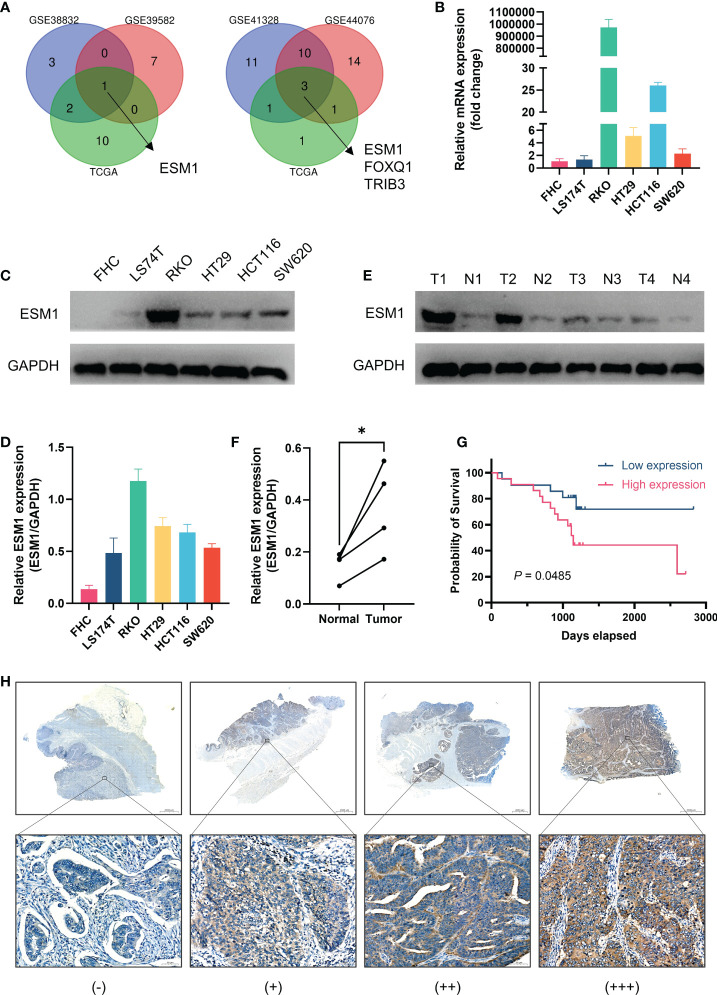
Validation of the role of ESM1 in CRC. **(A)** Venn diagram of prognosis-related genes in three datasets and screening characteristic genes in three datasets. **(B)** Validation of ESM1 gene expression in various cell lines by qPCR. **(C, D)** Western blot and the quantification of ESM1 protein expression in various cell lines. **(E, F)** Western blot and the quantification of ESM1 in CRC tumor tissues. *P < 0.05. **(G)** Kaplan-Meier survival analysis of ESM1 in different groups. **(H)** Representative images of ESM1 immunohistochemical staining (−: negative staining, +: weak positive, ++: medium positive, +++: strong positive. Scale bars: up, 2000 μm; below, 50 μm).

As illustrated by Venn diagrams in **Figure 7A**, the intersection of prognosis-related genes and different gene sets screening characteristic genes *via* machine learning algorithms in three datasets all pertained to one gene, ESM1, implicating a potentially essential role in CRC. ESM1 gene expression in various cell lines was inspected by qPCR, which showed that the expression of *ESM1* was significantly higher in tumor cells than in normal intestinal epithelial cell line FHC (**Figure 7B**). Consistent with this result, Western blot revealed a significantly higher expression of ESM1 protein in tumor cells than in normal intestinal epithelial cell line FHC as well (**Figures 7C, D**). Similarly, Western blot derived from patients’ tumors and paracancerous tissues also showed that ESM1 protein was higher in tumor tissue than in normal tissue (**Figures 7E, F**). We collected postoperative specimens from a total of 43 CRC patients to determine the expression of ESM1 in CRC tumor tissues. As shown in the **Figure 7H**, ESM1 was expressed variably in CRC. Intriguingly, patients with high ESM1 expression showed shorter disease-free survival (DFS) (median survival 1109 days vs. 1170 days, *P* = 0.0485, **Figure 7G**). As illustrated in **Table 2**, ESM1 expression was not associated with clinicopathological parameters such as age, sex, tumor position, pathological stage or histological differentiation (*P* > 0.05). This result was also corroborated by different datasets, which showed that high ESM1 was strongly associated with a poor prognosis of CRC (**Figure S3A**). The specificity and sensitivity of ESM1 to diagnose CRC were determined by the diagnostic ROC curves and the AUCs in GSE41328, GSE44076, GSE113513 and TCGA cohorts were 1.000, 0.993, 0.954, 0.999, respectively (**Figure S3B**). All these results revealed that ESM1 played an essential role in CRC and may serves as a reliable marker for the diagnosis and prognosis prediction of CRC.

**Table 2:** ESM1 expression characteristics in CRC patients.

Characteristics	Classification	Cases	Mean H-score	Standard deviation of H-score	*P* value
Age (year)	> 60	26	138.9	57.5	0.64
	≤ 60	17	147.5	59.6	
Sex	Male	31	144.3	55.5	0.72
	Female	12	137.2	65.4	
Position	Colon	6	114.2	55.4	0.76
	Rectum	37	145.6	58.2	
TNM stage	Unknown	12	150.8	65.9	0.30
	I	8	103.4	57.2	
	II	10	141.8	55.3	
	III	12	159.0	47.9	
	IV	1	156.1	0.0	
Differentiation	Unknown	31	143.0	57.4	0.90
	Poor	10	137.1	67.0	
	Moderate	2	157.1	1.4	

## Discussion

So far, the American Joint Committee on Cancer (AJCC) TNM staging system has been recognized as a credible tool for prognosis for CRC patients. However, TNM staging based on macroscopic information failed to reflect the tumor heterogeneity caused by molecular biological differences in CRC. Over the past decades, conventional chemotherapy regimens have not been satisfactory in treating patients with recurrent and refractory CRC (19). Even complete surgical treatment was performed, patients with CRC remain at a significant risk of recurrence and death (20). Thus, the development of relevant biomarkers with high prognostic value will facilitate better characterization of the transcriptional subtypes, mesenchymal and immune components of CRC, which is essential to further guide patient stratification for more accurate treatment guidance and prognostic assessment of outcomes. On the other hand, the identification of new molecular targets for immunotherapy holds great promise for the development of new targeted drugs and improved treatment strategies for CRC patients.

Prior studies that have noted the key role of MRGs or IRGs in CRC and several clinical indicators concerning immune or metabolism status have been developed for therapeutic guidance and prognostic assessment of CRC (9, 21–23). Nevertheless, patients with similar clinical characteristics remain highly heterogeneous at the microscopic molecular level, leading to significant differences in clinical outcomes. Integrated predictors that simultaneously respond to metabolic and immune status are more effective in improving prognostic value. Here in our study, we identified a combined immune and metabolism related prognostic signature that comprised 4 relevant genes based on the ranking of gene values. By effectively stratifying CRC patients, this signature served to predict patient prognosis and may be useful as an indicator for assessing response to immunotherapy. The effectiveness of the signature was validated by external validation datasets. In addition, an overexpressed gene, ESM1, was identified and its association with CRC prognosis was also verified. As far as we know, it is the first study to investigate the combined MRGs and IRGs-related prognostic signature in CRC.

As an essential component of immunotherapy, analysis of the tumor immune microenvironment (TIME) contributes to prediction of responsiveness to immunotherapy. Promising clinical results have been achieved in a variety of cancers by reprogramming the immunosuppressed state in tumors to an immune activated state (24). In our study, we explored the relationship between riskScore and immune cells in TIME and found that the high-risk group displayed a more abundant macrophage infiltration. This is consistent with previous studies demonstrating that tumor-associated macrophages were associated with poor prognosis in cancer patients (25). Furthermore, the high-risk group showed significantly higher stromal score compared with the low-risk group, indicated that CRC patients with high-risk scores have more abundant stromal components in TME, which may lead to a worse prognosis in the high-risk group due to greater susceptibility to metastasis. Compared to the traditional view with the classification of tumor immunophenotypes into “cold” and “hot”, our findings compared the differences in immune cell infiltration in high- and low-risk populations, which may provide a more precise model for immunotherapy of CRC.

Among the four genes included in the signature, NAT2 (N-acetyltransferase 2), as an important two-phase metabolic enzyme, exhibits evident genetic polymorphism and is considered to be strongly associated with CRC genetic susceptibility (26, 27). UGT2A3 (UDP glucuronosyltransferase 2 family, polypeptide A3) was found to be one of the molecules participating in the metabolism of xenobiotics by cytochrome P450, which has also been shown to be implicated in the metabolism of various anticancer agents. Associated with a better prognosis of CRC, upregulation of UGT2A3 expression was found to promote the metabolism of anticancer drugs and reduce chemical carcinogenesis (28). As a glycoprotein hormone, STC2 (Stanniocalcin 2) is associated with glutamine or glucose deprivation. Up-regulation of STC2 under hypoxia facilitates the adaptation of tumor cells to hypoxia and thus promotes tumor progression (29, 30). A more in-depth study of the mechanisms of these metabolic and immune-related genes is likely to provide new insights into the immunotherapy of CRC.

Another interesting point pertain to the role of ESM in CRC, as the intersection of different gene sets all converged to ESM1. Known as endocan, endothelial cell-specific molecule 1 (ESM1) is a secretory proteoglycan functioned as an important role in the exacerbation of inflammation and the proliferation, invasion and metastasis of tumors (31). Previous studies have demonstrated that ESM1 was increased in the tissues and serum of CRC patients and suggested that ESM1 could be a potential serum marker for early detection of CRC (32). It has been reported that high levels of serum ESM1 were significantly associated with poor overall survival in CRC and was an independent prognostic parameter for OS (33), which was consistent with our results in the mRNA level. ESM1 gene silencing significantly inhibited cell growth and metastatic process in CRC cells (31). This study was set out with the aim of assessing the importance of ESM1 in CRC. Similarly, our results suggested that ESM1 is highly expressed in both CRC tumor cells lines and tumor tissues, with this high expression indicating a poor prognosis for CRC. Other studies have also suggested an association of ESM1 with tumor angiogenesis and immunological characteristics (34, 35). Despite being in the theoretical and experimental stages, ESM1 will have infinite prospects in the future as a potential tumor marker for CRC and a novel target for cancer therapy.

In spite of these promising findings, several issues need to be addressed in the current study. Due to the long median survival of CRC patients and the fact that most of the patients enrolled for our immunohistochemical validation were hospitalized in 2019, we were unable to assess the OS of patients, which is one of our limitations. Another important point is that our result is based on RNA level rather than protein level, which may reduce the robustness of our conclusions. Next, gene expression characteristics are inevitably affected by sampling bias due to genetic heterogeneity within the tumor (36). In addition, further investigation of the underlying biological mechanisms of the signature is still needed in the forthcoming study.

### Conclusion

A novel combined MRGs and IRGs-related prognostic signature that could stratify CRC patients into low-and high- risk groups of unfavorable outcomes for survival, was identified and verified. This might help, to some extent, to individualized treatment and prognosis assessment of CRC patients. Similarly, the mining of the key gene provides a new perspective to explore the molecular mechanisms and targeted therapies of CRC.

## Data availability statement

The original contributions presented in the study are included in the article/**Supplementary Material**. Further inquiries can be directed to the corresponding authors.

## Ethics statement

The studies involving human participants were reviewed and approved by The Ethics Review Committee of the Fifth Affiliated Hospital of Sun Yat-sen University. Written informed consent for participation was not required for this study in accordance with the national legislation and the institutional requirements.

## Author contributions

YX and LW were responsible for the experiment operation. GZ was responsible for the statistical analysis. LW collected the experimental specimens. YX and GZ contributed to manuscript editing. YX and ML was responsible for the study design and manuscript review. All authors contributed to the article and approved the submitted version.

## Funding

This work was supported by the Natural Science Foundation of Guangdong Province (2022A1515012509).

## Conflict of interest

The authors declare that the research was conducted in the absence of any commercial or financial relationships that could be construed as a potential conflict of interest.

## Publisher’s note

All claims expressed in this article are solely those of the authors and do not necessarily represent those of their affiliated organizations, or those of the publisher, the editors and the reviewers. Any product that may be evaluated in this article, or claim that may be made by its manufacturer, is not guaranteed or endorsed by the publisher.

## References

[1] Bray F Ferlay J Soerjomataram I Siegel RL Torre LA Jemal A. Global cancer statistics 2018: GLOBOCAN estimates of incidence and mortality worldwide for 36 cancers in 185 countries. CA Cancer J Clin (2018) 68:394–424. doi: 10.3322/caac.21492 30207593

[2] Punt CJ Koopman M Vermeulen L. From tumour heterogeneity to advances in precision treatment of colorectal cancer. Nat Rev Clin Oncol (2017) 14:235–46. doi: 10.1038/nrclinonc.2016.171 27922044

[3] Dekker E Tanis PJ Vleugels J Kasi PM Wallace MB. Colorectal cancer. Lancet (2019) 394:1467–80. doi: 10.1016/S0140-6736(19)32319-0 31631858

[4] Guinney J Dienstmann R Wang X de Reynies A Schlicker A Soneson C. The consensus molecular subtypes of colorectal cancer. Nat Med (2015) 21:1350–6. doi: 10.1038/nm.3967 PMC4636487 26457759

[5] Dienstmann R Vermeulen L Guinney J Kopetz S Tejpar S Tabernero J. Consensus molecular subtypes and the evolution of precision medicine in colorectal cancer. Nat Rev Cancer (2017) 17:79–92. doi: 10.1038/nrc.2016.126 28050011

[6] Hu W Yang Y Qi L Chen J Ge W Zheng S. Subtyping of microsatellite instability-high colorectal cancer. Cell Commun Signal (2019) 17:79. doi: 10.1186/s12964-019-0397-4 31331345 PMC6647262

[7] Wu F Wang ZL Wang KY Li GZ Chai RC Liu YQ. Classification of diffuse lower-grade glioma based on immunological profiling. Mol Oncol (2020) 14:2081–95. doi: 10.1002/1878-0261.12707 PMC7463381 32392361

[8] Pavlova NN Thompson CB. The emerging hallmarks of cancer metabolism. Cell Metab (2016) 23:27–47. doi: 10.1016/j.cmet.2015.12.006 26771115 PMC4715268

[9] La Vecchia S Sebastian C. Metabolic pathways regulating colorectal cancer initiation and progression. Semin Cell Dev Biol (2020) 98:63–70. doi: 10.1016/j.semcdb.2019.05.018 31129171

[10] Brown RE Short SP Williams CS. Colorectal cancer and metabolism. Curr Colorectal Cancer Rep (2018) 14:226–41. doi: 10.1007/s11888-018-0420-y PMC6690608 31406492

[11] Martinez-Outschoorn UE Peiris-Pages M Pestell RG Sotgia F Lisanti MP. Cancer metabolism: A therapeutic perspective. Nat Rev Clin Oncol (2017) 14:11–31. doi: 10.1038/nrclinonc.2016.60 27141887

[12] Langfelder P Horvath S. WGCNA: An r package for weighted correlation network analysis. BMC Bioinf (2008) 9:559. doi: 10.1186/1471-2105-9-559 PMC2631488 19114008

[13] Ritchie ME Phipson B Wu D Hu Y Law CW Shi W. Limma powers differential expression analyses for RNA-sequencing and microarray studies. Nucleic Acids Res (2015) 43:e47. doi: 10.1093/nar/gkv007 25605792 PMC4402510

[14] Nunes C Rocha R Buzelin M Balabram D Foureaux F Porto S High agreement between whole slide imaging and optical microscopy for assessment of HER2 expression in breast cancer: Whole slide imaging for the assessment of HER2 expression. Pathol Res Pract (2014) 210: 713–8. doi: 10.1016/j.prp.2014.06.031 25091257

[15] Hong J Lin X Hu X Wu X Fang W. A five-gene signature for predicting the prognosis of colorectal cancer. Curr Gene Ther (2021) 21:280–9. doi: 10.2174/1566523220666201012151803 33045967

[16] Wang J Yu S Chen G Kang M Jin X Huang Y. A novel prognostic signature of immune-related genes for patients with colorectal cancer. J Cell Mol Med (2020) 24:8491–504. doi: 10.1111/jcmm.15443 PMC7412433 32564470

[17] Sun G Li Y Peng Y Lu D Zhang F Cui X. Identification of a five-gene signature with prognostic value in colorectal cancer. J Cell Physiol (2019) 234:3829–36. doi: 10.1002/jcp.27154 30132881

[18] Chen L Lu D Sun K Xu Y Hu P Li X. Identification of biomarkers associated with diagnosis and prognosis of colorectal cancer patients based on integrated bioinformatics analysis. Gene (2019) 692:119–25. doi: 10.1016/j.gene.2019.01.001 30654001

[19] Weng W Feng J Qin H Ma Y. Molecular therapy of colorectal cancer: Progress and future directions. Int J Cancer (2015) 136:493–502. doi: 10.1002/ijc.28722 24420815

[20] McQuade RM Stojanovska V Bornstein JC Nurgali K. Colorectal cancer chemotherapy: The evolution of treatment and new approaches. Curr Med Chem (2017) 24:1537–57. doi: 10.2174/0929867324666170111152436 28079003

[21] Zhang M Wang HZ Peng RY Xu F Wang F Zhao Q. Metabolism-associated molecular classification of colorectal cancer. Front Oncol (2020) 10:602498. doi: 10.3389/fonc.2020.602498 33344254 PMC7746835

[22] Jiang C Liu Y Wen S Xu C Gu L. In silico development and clinical validation of novel 8 gene signature based on lipid metabolism related genes in colon adenocarcinoma. Pharmacol Res (2021) 169:105644. doi: 10.1016/j.phrs.2021.105644 33940186

[23] Ke J Liu XH Jiang XF He Z Xiao J Zheng B. Immune-related gene signature in predicting prognosis of early-stage colorectal cancer patients. Eur J Surg Oncol (2020) 46:e62–70. doi: 10.1016/j.ejso.2020.08.008 32863096

[24] Ohe C Yoshida T Ikeda J Tsuzuki T Ohashi R Ohsugi H. Histologic-based tumor-associated immune cells status in clear cell renal cell carcinoma correlates with gene signatures related to cancer immunity and clinical outcomes. Biomedicines (2022) 10 (2):323. doi: 10.3390/biomedicines10020323 35203532 PMC8869140

[25] Zhou SL Zhou ZJ Hu ZQ Huang XW Wang Z Chen EB. Tumor-associated neutrophils recruit macrophages and T-regulatory cells to promote progression of hepatocellular carcinoma and resistance to sorafenib. Gastroenterology (2016) 150:1646–58. doi: 10.1053/j.gastro.2016.02.040 26924089

[26] Tamer L Ercan B Ates NA Degirmenci U Unlu A Ates C. N-acetyltransferase 2 gene polymorphism in patients with colorectal carcinoma. Cell Biochem Funct (2006) 24:131–5. doi: 10.1002/cbf.1191 15617035

[27] Liu H Fu ZX Wang CY Qian J Xing L Liu YW. A meta-analysis of the relationship between NAT2 polymorphism and colorectal cancer susceptibility. Med (Kaunas) (2012) 48:117–31. doi: 10.3390/medicina48030017 22588343

[28] Pang B Xu X Lu Y Jin H Yang R Jiang C. Prediction of new targets and mechanisms for quercetin in the treatment of pancreatic cancer, colon cancer, and rectal cancer. Food Funct (2019) 10:5339–49. doi: 10.1039/c9fo01168d 31393490

[29] Qie S Liang D Yin C Gu W Meng M Wang C. Glutamine depletion and glucose depletion trigger growth inhibition *via* distinctive gene expression reprogramming. Cell Cycle (2012) 11:3679–90. doi: 10.4161/cc.21944 PMC3478318 22935705

[30] Zhang C Chen S Ma X Yang Q Su F Shu X. Upregulation of STC2 in colorectal cancer and its clinicopathological significance. Onco Targets Ther (2019) 12:1249–58. doi: 10.2147/OTT.S191609 PMC6389002 30863092

[31] Kang YH Ji NY Han SR Lee CI Kim JW Yeom YI. ESM-1 regulates cell growth and metastatic process through activation of NF-kappaB in colorectal cancer. Cell Signal (2012) 24:1940–9. doi: 10.1016/j.cellsig.2012.06.004 22735811

[32] Ji NY Kim YH Jang YJ Kang YH Lee CI Kim JW. Identification of endothelial cell-specific molecule-1 as a potential serum marker for colorectal cancer. Cancer Sci (2010) 101:2248–53. doi: 10.1111/j.1349-7006.2010.01665.x PMC11158300 20735430

[33] Jiang H Fu XG Chen YT. Serum level of endothelial cell-specific molecule-1 and prognosis of colorectal cancer. Genet Mol Res (2015) 14:5519–26. doi: 10.4238/2015.May.25.3 26125749

[34] Li S Liang P Zhao Y Li X Hu Y Wu W. Detection and mechanism of action of ESM-1 in rat kidney transplantation under various immune states. Cell Immunol (2013) 283:31–7. doi: 10.1016/j.cellimm.2013.05.003 23850961

[35] Ziol M Sutton A Calderaro J Barget N Aout M Leroy V. ESM-1 expression in stromal cells is predictive of recurrence after radiofrequency ablation in early hepatocellular carcinoma. J Hepatol (2013) 59:1264–70. doi: 10.1016/j.jhep.2013.07.030 23928407

[36] Gerlinger M Rowan AJ Horswell S Math M Larkin J Endesfelder D. Intratumor heterogeneity and branched evolution revealed by multiregion sequencing. N Engl J Med (2012) 366:883–92. doi: 10.1056/NEJMoa1113205 PMC4878653 22397650

