# Supplementary material for: Exploration and validation of a combined immune and metabolism gene signature for prognosis prediction of colorectal cancer
Source: Front Endocrinol (Lausanne). 2022 Nov 28;13:1069528. doi: 10.3389/fendo.2022.1069528 (PMC9742469; doi:10.3389/fendo.2022.1069528)
Supplement: Supplementary file 1 [file DataSheet_1.docx]

# Exploration and validation of a combined immune and metabolism gene signature for prognosis prediction of colorectal cancer

**Yitai Xiao^1^†*, Guixiong Zhang^2^†, Lizhu Wang^3^†, Mingzhu Liang^3^***

^1^Guangdong Provincial Key Laboratory of Biomedical Imaging and Guangdong Provincial Engineering Research Center of Molecular Imaging, The Fifth Affiliated Hospital, Sun Yat-sen University, Zhuhai, Guangdong Province, P. R. China 519000.

^2^Department of Interventional Oncology, The First Affiliated Hospital, Sun Yat-Sen University, Guangzhou, Guangdong Province, P. R. China 510080.

^3^Department of Radiology, The Fifth Affiliated Hospital of Sun Yat-sen University, Zhuhai, P. R. China 519000.

*Corresponding authors at: The Fifth Affiliated Hospital, Sun Yat-sen University, Zhuhai, Guangdong Province, P. R. China 519000.

E-mail addresses: xiaoyt3@mail2.sysu.edu.cn (Yitai Xiao), liangmzh5@mail.sysu.edu.cn (Mingzhu Liang)

†These authors contributed equally to this work.


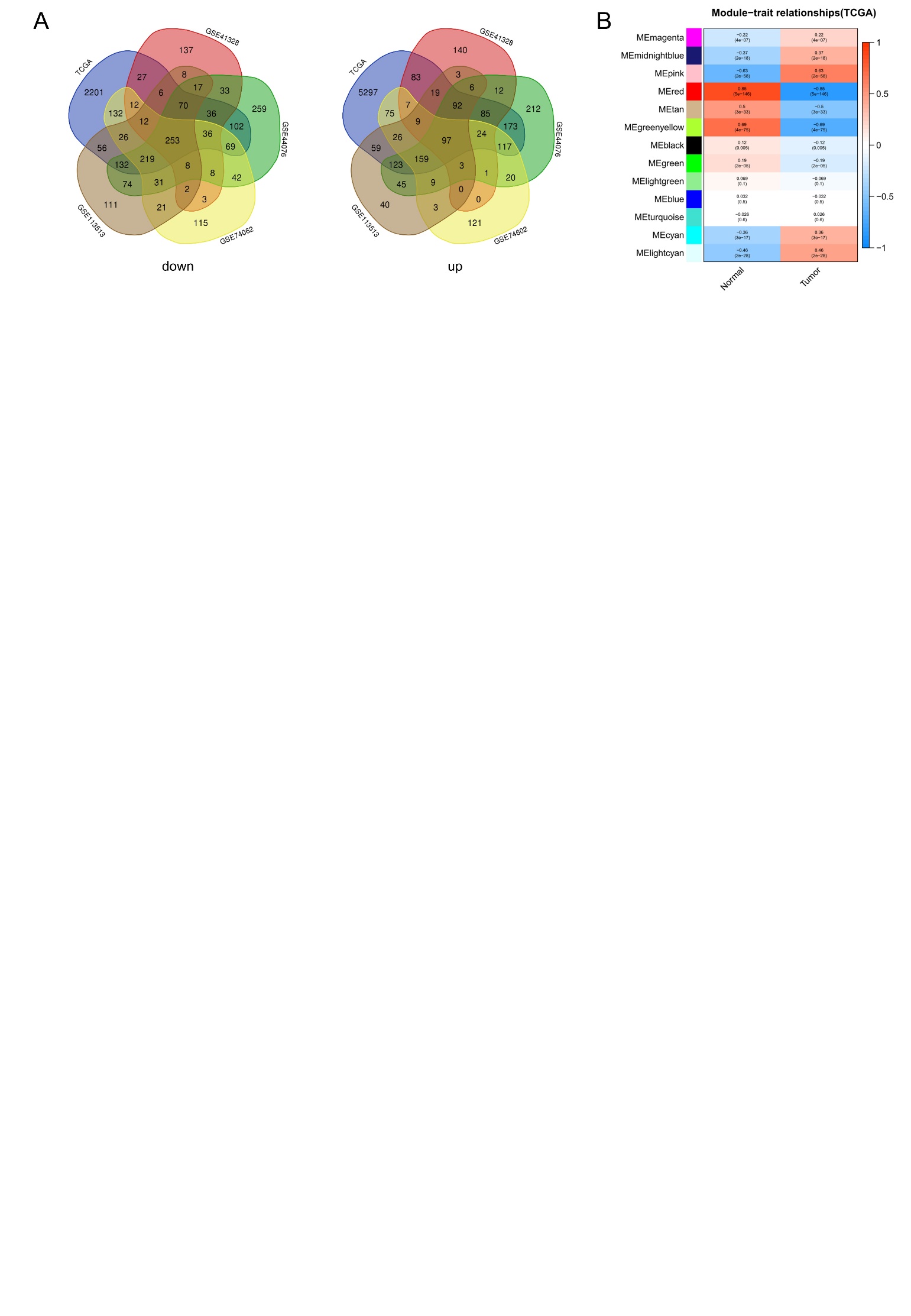


**Figure S1** (A) Venn diagram of the downregulated and upregulated differentially expressed genes (DEGs) among CRC patients. (B) Relationship between the modules and the tumor or nomal. Different color blocks represent different clusters of genes. Color shades represent the correlation.


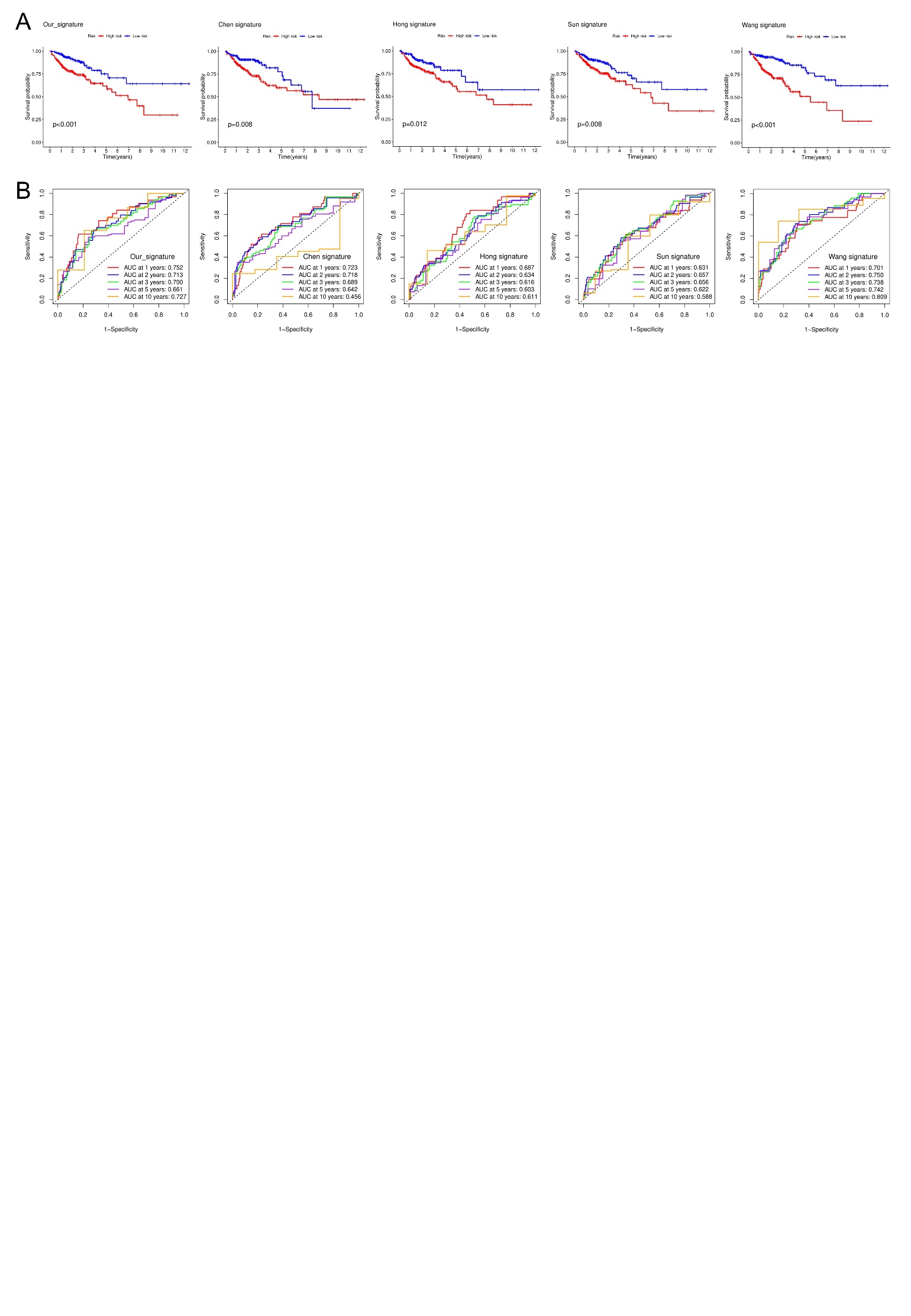


**Figure S2** (A) Kaplan-Meier OS curves of our signatures and other existing signatures. (B) ROC curves of our signatures and other existing signatures.


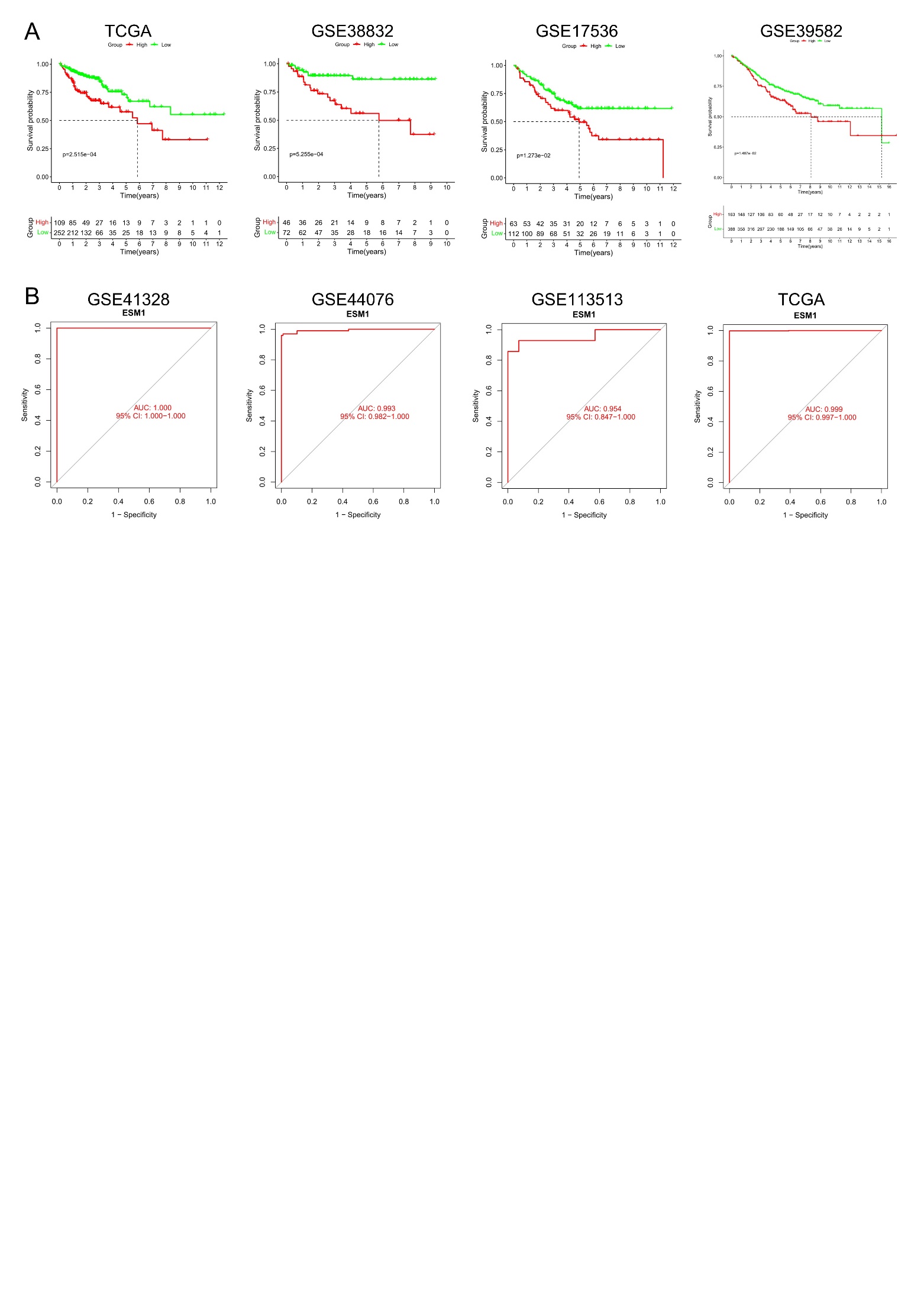


**Figure S3** (A) Kaplan-Meier curves of ESM1 in different datasets. (B) ROC curves of ESM1 to diagnose CRC in different datasets

.
